# Endoscopic Evacuation of Basal Ganglia Hemorrhage via Keyhole Approach Using an Adjustable Cannula in Comparison with Craniotomy

**DOI:** 10.1155/2014/898762

**Published:** 2014-05-18

**Authors:** Heng-Zhu Zhang, Yu-Ping Li, Zheng-cun Yan, Xing-dong Wang, Lei She, Xiao-dong Wang, Lun Dong

**Affiliations:** Department of Neurosurgery, Clinical Medical College of Yangzhou University, 98 Nan Tong West Road, Yangzhou, Jiangsu 225001, China

## Abstract

Neuroendoscopic (NE) surgery as a minimal invasive treatment for basal ganglia hemorrhage is a promising approach. The present study aims to evaluate the efficacy and safety of NE approach using an adjustable cannula to treat basal ganglia hemorrhage. In this study, we analysed the clinical and radiographic outcomes between NE group (21 cases) and craniotomy group (30 cases). The results indicated that NE surgery might be an effective and safe approach for basal ganglia haemorrhage, and it is also suggested that NE approach may improve good functional recovery. However, NE approach only suits the selected patient, and the usefulness of NE approach needs further randomized controlled trials (RCTs) to evaluate.

## 1. Introduction

Worldwide, intracerebral hemorrhage (ICH) is a major cause of morbidity and mortality [1]. It is the second most common form of stroke, accounting for 13–20% of first-time strokes [2–4]. Basal ganglia haemorrhage is a common type of ICH, and it is a life-threatening condition that may result in a series of complications, including hematoma expansion, severe brainstem compression, acute hydrocephalus, increased intracranial pressure, seizures, fever, and infections [5]. To avoid these complications, patients usually need urgent surgical treatment.

Surgical management on basal ganglia hemorrhage has unique advantages as it can remove the hematoma effectively and decrease intracranial pressure and the incidence of complications. In recent years, basal ganglia haemorrhage has been commonly treated using conventional craniotomy, which has high mortality (22%–36%) and poor hematoma evacuation rate (44%–74%), as shown in some studies [6, 7]. A large randomized clinical trial [8] did not show significant benefits of surgery compared to conservative medical treatment. Furthermore, the AHA/ASA Guidelines for Managing Spontaneous Intracerebral Hemorrhage suggested that the effectiveness of the craniotomy approach for basal ganglia haemorrhage is still uncertain [9].

More recently, with improvements in neuroendoscopic (NE) techniques, basal ganglia hemorrhage has begun to be approached using the technique of NE [10]. Some studies indicated that the endoscope-assisted keyhole approach might be an efficiency, safety, and minimal invasiveness surgical intervention [11, 12]. This study presented a new adjustable cannula application in NE operation via keyhole approach. At the Clinical Medical College of Yangzhou University, we have been applying the minimally invasive technique of NE approach to evacuate basal ganglia haemorrhage since 2011. Here, we compared the clinical and radiological outcomes of two intervention groups (NE group versus traditional craniotomy group). The goals of this study are to evaluate the effectiveness and safety of the NE approach to basal ganglia haemorrhage comparing with the craniotomy and to analyse the influences behind the surgical outcomes.

## 2. Subjects and Methods

This study was conducted in basal ganglia haemorrhage patients who had undergone NE or craniotomy in our department since June 2011. Fifty-one patients were randomly divided into 2 groups, the NE group (21 cases) and the traditional craniotomy group (30 cases), that underwent NE surgery or craniotomy, respectively.

All the patients were screened and enrolled: (a) ICH occurred <24 h, (b) it was diagnosed by both a clinical and a brain CT examination, (c) hematoma volume was >25 mL, and (d) there were significant neurological symptoms due to the acute hematoma. The exclusion criteria were any clinical signs of herniation, ICH located in the posterior cranial fossa or extension of the ICH into the brainstem, and any patients with tumor, coagulopathy, vascular lesion, or aneurysm diagnosed by CT scan, CT angiography, or coagulation function tests.

Clinical data were collected for all cases during preceding preventative treatment. CT scans were performed to calculate the intracerebral hematoma volumes and to provide a precise definition of the variations in the preoperative anatomical and pathological structures. The ICH hematoma volume was calculated from the brain CT using the TADA formula [13] (*V* = length width × thickness/2). The data obtained from these patients were analysed with respect to the clinical and radiographical outcomes, including gender, age, hematoma volume, Glasgow Coma Scale (GCS) on admission, physiological data, hematoma evacuation rate, operation time, and complications.

We also assessed neurological function recovery. All the patients had undergone neurological examinations at admission, after operation, and at each follow-up appointment. The following neurological treatment details were collected: (1) the GCS on the third postoperative day and at discharge; (2) the Glasgow Outcome Scale (GOS) at discharge and at follow-up examinations; (3) the modified Rankin Scale (mRS) on admission, on discharge, and during follow-up examinations conducted 6 months after surgery, and (4) good functional outcome (GFO), which is defined as a patient being able to care for himself, corresponding to mRS of 0, 1, 2, or 3 and GOS of 4 or 5.

### 2.1. Surgical Procedure of NE Approach

**Figure 1:**
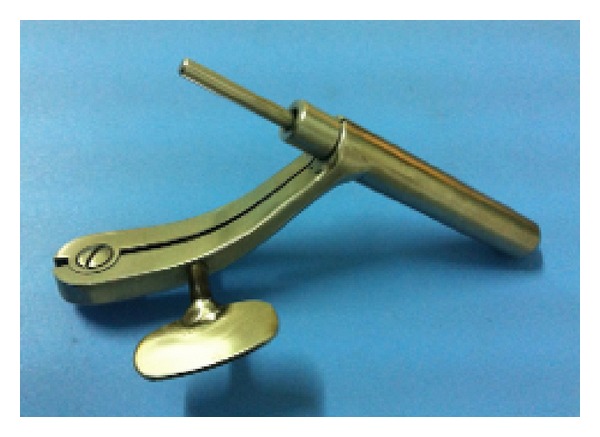
Photograph of an adjustable cannula with metal stylet.

Evacuation of basal ganglia hemorrhage was performed through a rigid NE with an adjustable cannula (developed by our surgical team, as shown in Figure 1). The surgical procedure was conducted under general anesthesia. Linear skin incisions were 4.0 to 5.0 cm long over the transtemporal approach in basal ganglia haemorrhage. The bone windows were 1.5 cm to 3.0 cm in diameter. The adjustable cannula was inserted followed by stylet application to confirm the location of hematoma. Then the NE, suction unit, or bipolar coagulator was introduced into cannula to evacuate the hematoma. The central part of the hematoma was evacuated using 2.0 to 4.0 mm suction. During the operation, obvious bleeding was stopped using the bipolar coagulator under low output power (4 to 8 Watts). When the evacuation was complete, saline was irrigated to locate any points of bleeding. Then, homeostatic fibers were used to cover the surface of the hematoma cavity, and the control valve is switched to close the sheath canal again, regaining the bullet shape. Finally the adjustable cannula is pulled out slowly and the operation is finished.

After the operation, all patients were transferred to the neurointensive care unit (NICU) for a few days until their condition began to stabilise. Repetitive CT scans were performed within 24 h, on the third day, and at any point of follow-up.

### 2.2. Statistical Analysis

All statistical analyses were performed using SPSS 19.0. A probability value of less than 0.05 was considered statistically significant. The data regarding age, hematoma volume, operation time, GOS score, GCS score, and mRS score were expressed in terms of mean values with corresponding standard deviations. An independent 2-sample *t*-test was employed for comparison of the two intervention groups.

### 2.3. Illustrative Case-A Classical Case of Basal Ganglion Hematoma

**Figure 2:**
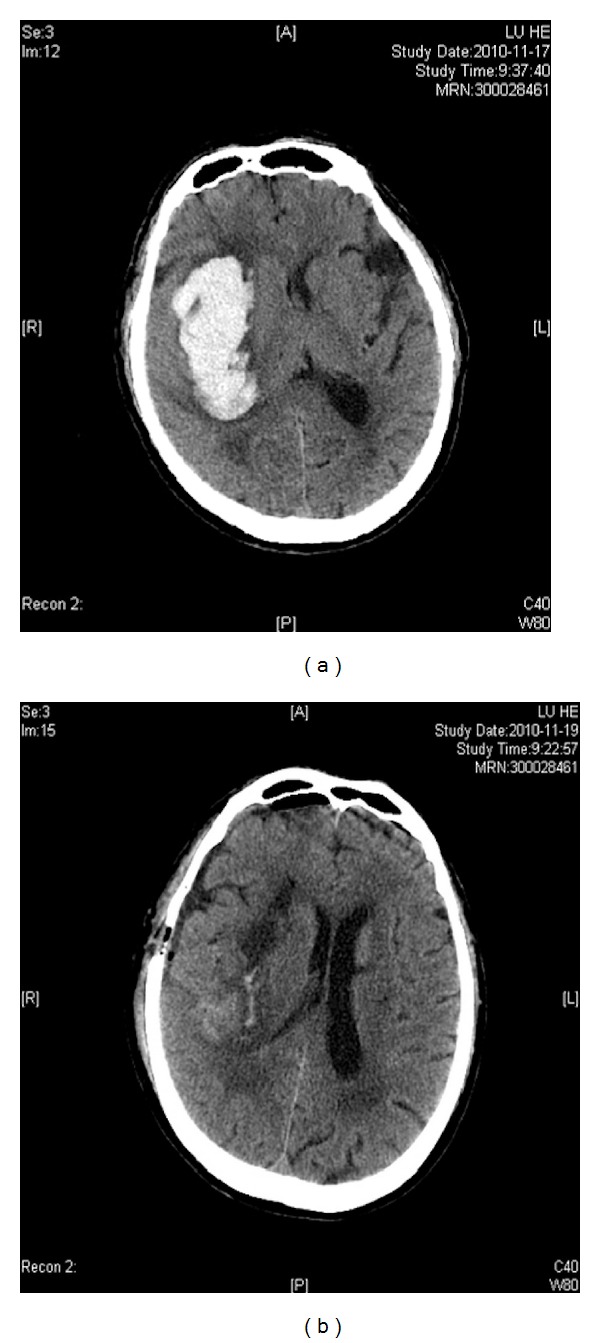
The CT scan results of illustrative case. (a) The CT scan showed preoperational volume of hematoma. (b) The CT scan revealed hematoma volume 24 hours after surgery.

**Figure 3:**
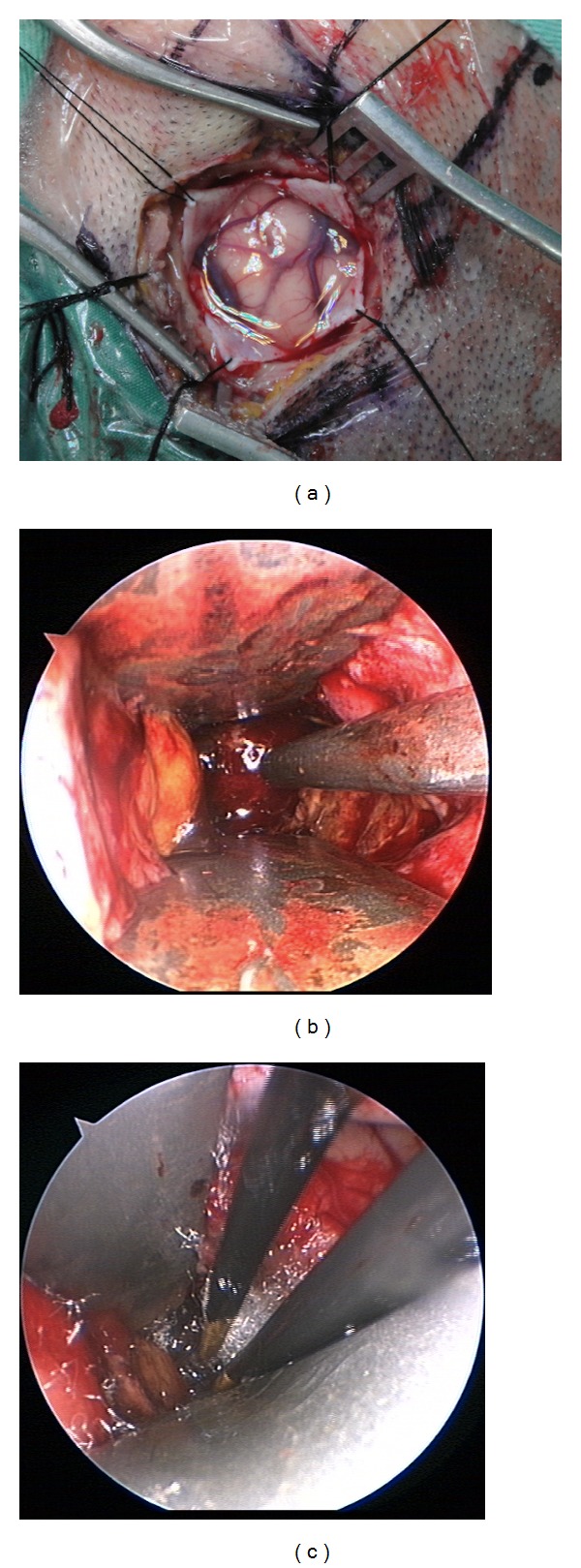
Using adjustable cannula in neuroendoscopic surgery. (a) A 4 cm long linear skin incision is shown and 2.5 cm keyhole craniotomy in diameter was used in patient. (b) The suction unit was applied to evacuate hematoma. (c) The bipolar coagulator hemostasis was applied in bleeding vessels.

A 76-year-old man was transferred to our hospital due to a right basal ganglia cerebral hemorrhage. On admission, his GCS score was 9. A brain CT scan revealed a right-lateral thalamic hemorrhage with moderate mass effect (Figure 2(a)). The volume of the hematoma was estimated to be 64.8 mL. We applied the temporal approach on this patient. The linear skin incision was 4 cm long, and the bone hole was 2.5 cm in diameter. Postoperative computed tomographic scanning revealed almost complete removal of the thalamic hematoma (Figure 2(b)). The hematoma evacuation rate was 93.3%. The patient regained consciousness 1 week after surgery and could work independently at discharge. Six months later he had a GOS of 4 and a mRS score of 2. The intraoperative photo was shown in Figure 3.

## 3. Results

Between June 2011 and July 2012, 176 patients with basal ganglia hemorrhage were admitted to the Clinical Medical College of Yangzhou University. According to the inclusion and exclusion criteria mentioned above, a total of 51 cases were included, of which 21 involved patients who underwent NE approach and 30 underwent a traditional craniotomy. This study included 38 men and 13 women, and the mean patient age was 60.68 years (range from 23 to 70). All patients underwent surgery within 24 hours of ictus, and 19 patients (37.2%) underwent surgery within 12 hours. The mean clinical follow-up was 8.2 months (ranging from 6 to 20 months), and no patient was lost to follow-up. There were no statistically significant differences in the baseline characteristics of each group, including age, sex, admission GCS score, admission mRS score, history of hypertension, and time between symptom onset and surgery, as shown in Table 1.

### 3.1. Early Clinical and Neurological Functional Outcomes

There was a statistical difference in the hematoma evacuation rate between the NE and craniotomy groups (90.11% ± 7.27% in the NE group versus 85.37% ± 6.78% in the craniotomy group; *P* = 0.02). Operation time was 76.48 ± 14.92 min in the NE group, significantly shorter than 175.15 ± 26.13 min in the craniotomy group (*P* < 0.00001). The mean NICU stay was 6.5 days in the NE group and 11.2 days in the traditional craniotomy group (*P* = 0.005).

### 3.2. Follow-Up Clinical and Functional Outcomes

No patients died in the NE group, and three died in the traditional craniotomy group, but there was no significant difference in the mortality of the two groups (*P* = 0.27). There was 1 case of rebleeding in the endoscopy group and 3 cases in the craniotomy group, but there were no significant differences in the rebleeding rate between the two groups (4.76% versus 10.0%; *P* = 0.50). In terms of infectious complications which included pneumonia and wound infection, there was 1 case of pneumonia and 1 case of wound infection in the endoscopy group and 9 cases of pneumonia and 2 cases of wound infection in the craniotomy group, with a significant difference in the incidence of infectious complications (9.52% versus 36.67%; *P* = 0.04), as shown in Table 2.

With respect to long-term neurological functional outcomes, there were no significant differences in the mean GOS scores (*P* = 0.07), GCS scores (*P* = 0.08), and mRS scores (*P* = 0.49) between the two intervention groups. However, the GFO was 52.38% in the NE group, slightly higher than 13.33% in the control group (*P* = 0.04).

## 4. Discussion

The basal ganglia hemorrhage is a common neurological disease with historically poor prognosis and outcomes [15]. Virtually all aspects of the management of basal ganglia hemorrhage are still not uniformly agreed upon [16]. The prognosis is influenced by several factors, including the origin of bleeding, initial GCS score, and hematoma volume. During the first 12 hours after onset, the intracranial pressure (ICP) can increase suddenly due to a mass effect associated with hematoma volume [17]. This factor may cause a significant reduction in cerebral blood flow to the brain tissue surrounding the hematoma, potentially leading to ischemia. Therefore, hematoma evacuation is a main target of surgical treatment. The early craniotomy surgery could immediate removal of the hematoma, a dramatic reduction of ICP, relief of cerebral edema, improvement in local blood circulation, and a reduction in mortality [18]. However, early methods of craniotomy failed to protect the still functional brain tissue surrounding the hematoma and caused too much damage. A Cochrane systematic review [19] revealed that the use of traditional craniotomy for the treatment of ICH remains controversial.

Advances in neuroimaging, together with rising interest in minimally invasive techniques, have resulted in the establishment of modern neuroendoscopy [20]. NE surgery has many advantages, such as minimally invasive, high evacuation rate, low incidence of complication, better protection of brain tissue, and less surgery related injuries [21]. In recent decades, some studies of ICH evacuation using the NE approach have placed great emphasis on protection of the surrounding brain regions and demonstrated high evacuation rate (ranging from 83.4% to 99%) [14, 22, 23]. Results of the radiographical outcomes in our study showed that the hematoma evacuation rate in the NE group was higher than in the traditional craniotomy group (*P* < 0.05). In this present study, we applied NE through an adjustable cannel to treat basal ganglia hemorrhage. This new application has several benefits. Firstly, the end of auxiliary sheath is in bullet shape, which could protect the brain tissues during operation. Secondly, the precise scales marked on the sheath canal could help surgeon to reach the predicted depth with accuracy. In association with the craniocerebral lesion or the hematoma exacted by sheath canal, the position of brain lesions or hematoma would be determined precisely. Thirdly, the control valve is rotated, being wide enough to form a channel to expose brain lesions to a large extent. Therefore the surgeon could work more flexibly under endoscopy. Fourthly, the bolt of auxiliary sheath is connected to the fixed operation device, which is beneficial for the removal of cerebellar hematoma or brain lesions under endoscopy. Fifthly, the material of auxiliary sheath is titanium alloy that is light weighted and tough enough to be adopted in this case. Last but not least, this application is designed carefully but not complicated, which makes it easily to be manufactured.

In terms of incidence of complications, the study from Nagasaka [24] indicated that endoscope-assisted ICH evacuation was associated with a minimal rebleeding rate (0%–3.3%) when compared to the traditional craniotomy approach (5%–10%). When we applied NE in the treatment of basal ganglia haemorrhage, the follow-up outcomes revealed lower rebleeding rate in the NE group than in the control group (4.76% versus 10%) but this result did not show statistical significance (*P* = 0.50). However, we found that this approach permitted direct identification of the bleeding points and permitted coagulation of the responsible vessels under endoscopic visualisation without overstretching the brain tissue. The incidence of infectious complications in the NE group was low, because of milder brain injury, shorter skin incisions, and shorter operation times. The reasons for these benefits are multiple and include the following. (a) The first reason is adequate exposure of the hematoma and adjacent vessels. The NE provides enough space and visibility to manage intraoperative bleeding. There was excellent visual quality in the deep and narrow fields when using adjustable cannula. In this study, the offending vessel was found in more than half of the cases. We used the bipolar coagulator on the active bleeding point and gelatin sponge compression hemostasis on the minor bleeding. It was easy to find the bleeding point and to ensure effective hemostasis. (b) In this study, we selected short, direct, and precise routes to the hematomas and deep lesions without manipulating or exposing the unaffected areas, which are essential for keyhole surgery. (c) The incidence of complications (rebleeding rate) related to the endoscopic approach was lower than those achieved by traditional craniotomy approach. (d) The fourth reason is short NICU stays and operative time. The results showed that patients in the NE group stayed in the NICU for a shorter time (*P* = 0.04) than patients in the control group. The NE approach did not require drainage and avoided the ongoing inflammatory response caused by blood and its breakdown products, resulting in faster recovery. The mean operative time resulting from our study showed shorter operation times when using the NE approach (*P* < 0.00001). (e) This research used self-made adjustable cannula, which has several advantages in protection of the brain tissues during operation and secondly helping the surgeon to reach the predicted depth with accuracy.

We also summarised the experience of endoscopic surgery for the treatment of SICH. (a) Appropriate operative routes should be chosen carefully and this is the key for success. The location of bone hole should be accessed by considering the location of hematoma and important blood vessels as well as functional areas [25]. As is known, frontal or temporal lobe is usually chosen as the entry site of operations to treat hematoma in basal ganglion [26]. Entry in temporal lobe is selected for endoscopic removal of basal ganglia hematoma in the nondominant hemisphere, whereas frontal lobe entry is chosen for hematoma in the dominant hemisphere. If the hematoma volume exceeds 50 mL, better prognosis could be achieved if the frontal lobe entry is performed compared to temporal lobe entry. (b) The endoscopic removal of intracranial hematoma is preceded within the cavity, which is good for minimising the effects to normal brain tissues and surrounding damaged brain tissues. This is also beneficial for the protection of rehemorrhage and hemostasis. The hematoma should be evacuated as clear as possible in principle. However, in case of large solid hematoma, biopsy forceps are used to mince the hematoma firstly and then evacuation of hematoma is performed. If the bleeding is suspected to occur primarily around blood vessels, the removal of hematoma has to avoid dragging movements that could cause massive bleeding. Such hematoma could be retained if necessary to lower the possibility of rehaemorrhage [27]. When the ICH patients have intraventricular hemorrhage, the external ventricular drainage should be performed. It is aiming to ensure that the cerebrospinal fluid circulation is free and functions normally in the case of onset of acute hydrocephaly after surgery. (c) Procedure after hemorrhage: the hemostasis has to be clear and thorough under endoscopy. A clear vision under endoscopy is the key for operation. The hemorrhage in arteriola must be observed with the help of suction apparatus and then coagulation hemostasis would be applied. If the bleeding point is not clear, cauterisation on the cavity wall of hematoma should be avoided. (d) The entry of endoscopy should be slow and gentle so as to obtain clear observation. Thus, the bleeding would be seen and dealt with in time. After endoscopic removal of intracranial hematoma, artificial cerebrospinal fluid would be used to fill the empty cavity and wash the remnants of hematoma, resulting in a clear vision under endoscopy. Moreover, artificial cerebrospinal fluid helps in supporting the remnant cavity, protecting the brain tissues from collapsing. Such brain damage could cause avulsion of bridging vein as well as subdural hematoma after surgery [28].

In conclusion, applying the NE approach using adjustable cannula might be an effective and safe approach for basal ganglia hemorrhage. The results of this study suggested that NE approach may improve good functional recovery. However, NE approach only suits the selected patient, and our study has several potential limitations. This study involved a relatively small patient group. It is our hope that a larger randomised control trial will be performed to evaluate the usefulness of the NE approach for the treatment of basal ganglia haemorrhage.

## Figures and Tables

**Table 1:** Clinical characteristics of included patients between NE group and the traditional craniotomy group.

	NE group	Craniotomy group	*P*
Cases (*n*)	21	30	—
Male/female (*n*)	16/5	22/8	0.82
Age (years)	59.90 ± 12.85	61.45 ± 9.25	0.64
Preoperation hematoma volume (mL)	58.28 ± 18.84	62.16 ± 15.62	0.44
GCS score	9.19 ± 3.76	8.37 ± 2.39	0.38
mRS score	3.57 ± 1.66	3.88 ± 2.14	0.56
History of hypertension (*n*/%)	15/76.19%	23/73.33%	0.67
Time between symptom onset and surgery, 12 h	9	10	0.89

*P* < 0.05 showing statistically significant differences.

**Table 2:** Clinical findings and functional outcomes in included patients.

Variable		NE group	Craniotomy group	*P* (95% CIs)
Hematoma evacuation rate (%)		90.11 ± 7.27	85.37 ± 6.78	0.02*
Operating time (min)		76.48 ± 14.92	175.15 ± 26.13	<0.00001*
Mortality (*n*/%)		—	3/10.0	0.27
Rebleeding rate (*n*/%)		1/4.76	3/10.0	0.50
Infection rate (*n*/%)		2/9.52	11/36.67	0.04*
Mean GOS score	6th month	3.61 ± 0.92	3.05 ± 1.26	0.07
Mean mRS score	Discharge	2.85 ± 1.88	2.96 ± 1.65	0.83
	6th month	2.33 ± 1.83	2.67 ± 1.54	0.49
Mean GCS score	3 days after surgery	9.71 ± 2.64	9.03 ± 2.85	0.38
	Discharge	11.61 ± 2.87	10.25 ± 2.45	0.08
Good functional outcome (*n*/%)		11/52.38	4/13.33	0.04*
NICU (d)		6.5	11.2	0.005*

*P < 0.05 showing statistically significant difference.

Good functional outcome (GFO) is defined as a patient being able to care for him/herself, corresponding to a modified Rankin Scale (mRS) of 0, 1, 2, or 3, a Glasgow Outcome Scale (GOS) of 4 or 5, or activities of daily living (ADL) score [14] of 1, 2, or 3.
